# Effects of Plant Extracts on Human Health

**DOI:** 10.3390/nu17071229

**Published:** 2025-04-01

**Authors:** Fátima Regina Mena Barreto Silva, Marcela Aragón Novoa

**Affiliations:** 1 Instituto de Bioeletricidade Celular (IBIOCEL): Ciência & Saúde, Departamento de Bioquímica, Universidade Federal de Santa Catarina, Florianopólis 88049-900, SC, Brazil; 2 Departamento de Farmacia, Universidad Nacional de Colombia, Av. Carrera 30 # 45-03 Edif 450, Bogota 111321, Colombia

The extracts of plants exhibit a variety of bioactive compounds (polyphenols, carotenoids, fibers, essential oils, polysaccharides, alkaloids, and proteins), with the majority having biological effects, but their mechanisms of action are still unknown. Furthermore, in this kind of sample, the challenge is choosing between the variability of compounds in the crude extract, which exhibit activity in a selected cellular target for a specific disease of interest. This Special Issue is based on medicinal plants and explores the potential effect of their extracts and/or their compounds in specific experimental models of diseases. The effect of curcumin on hexokinase 2 was studied through cellular adaptation to lactic acidosis on intracellular energy metabolism and sensitivity to docetaxel in prostate carcinoma cells. From this set of data, it was observed that metabolic plasticity through enhanced glycolysis in lactate-acclimated prostatic cells may be one pathway for docetaxel resistance; therefore, targeting glycolysis using curcumin may provide potential for drug development and contribute to advancements in therapy for prostate cancer patients. Additionally, in an ex vivo model using C57BL/6 male mouse prostate and a prostate cancer cell line, the protective effects of aged black garlic water extract (ABGE) were evidenced; it exhibited anti-inflammatory and antioxidant effects in these preclinical models, partly attributed to catechin and garlic acid. In this ex vivo model, ABGE reduced the gene expression of COX-2, NF-κB, TNF-α, and IL-6. For in vitro studies, ABGE inhibited cell proliferation, colony and tumor sphere formation, and the cell migration of prostate cancer cells, emphasizing its potential therapeutic effects.

Different kinds of natural compounds act to inhibit inflammatory responses, such as, for example, the natural alkaloid, tryptanthrin. This compound significantly reduced the mRNA expression of oncostatin M (anti-inflammatory cytokin) in the granulocyte–macrophage, colony-stimulating factor (GM-CSF)-stimulated, neutrophil-like dHL-60 cells and inhibited the phosphorylation of phosphatidylinositol 3-kinase (PI3K), AKT, and nuclear factor (NF)-κB. In addition, tryptanthrin decreased the oncostatin M production in GM-CSF-stimulated neutrophils from mouse bone marrow. The potential anti-inflammatory effect of Colchicum luteum hydroethanolic extract (CLHE) compared with non-steroidal, anti-inflammatory drugs focused in on COX-2, and TNFα inhibition was studied in a collagen-induced arthritic mice model. Molecular docking identified that the two main non-toxic compounds possessed strong binding affinities to COX-2 and TNG-α. Therefore, as a whole, these data suggest that CLHE is a safer, anti-inflammatory, and multi-targeted alternative to NSAIDs for rheumatoid arthritis treatment. Another extract (*Lysimachia mauritiana* Lam. extract) alleviates the airway inflammation induced by particulate matter and diesel exhaust particles in mice. *Lysimachia mauritiana* alleviated the accumulation of neutrophils and the number of inflammatory cells in the lungs and the bronchoalveolar lavage fluid of the mice exposed to particulate matter with a diameter of less than 10 µm, and also reduced the release of inflammatory mediators in the bronchoalveolar fluid and lungs. In addition, this extract inhibited MAPK and NF-κB signaling in the lungs. Altogether, this study indicates that this extract may be a promising therapeutic agent against inflammatory respiratory diseases.

For hyperuricemia, a metabolic disorder in the purine of the body discussed here, *Portulaca oleracea* (PO) inhibits xanthine oxidase activity through the effect of berberine and stachydrine isolated and purified from this plant and, therefore, reduces uric acid production. Furthermore, PO may reduce the body’s reabsorption of urate and aid in its excretion out of the body by inhibiting the urate transporter proteins (GLUT9, URAT1) and promoting the high expression of urate excretory protein (ABCG2). The results of histology showed that, compared with the positive drug (allopurinol and benzbromarone) group, there was no obvious renal injury in the middle- and high-dose groups of PO extract. On the whole, PO represents a potential functional food for the treatment of hyperuricemia.

Another interesting point were the studies related to hearing loss. The variable etiology for sensorineural hearing loss includes noises above decibels considered bearable for the ear, ototoxic agents, and aging, which can damage the inner ear or the auditory nerve. Studies on *Castanopsis echinocarpa* explore this medicinal plant as a potential therapeutic agent for hearing loss via critical neuronal gene regulation. In addition, in vivo experiments using zebrafish and mouse revealed otic hair cell protection in zebrafish, improved auditory function, and the protection of cochlear sensory cells in a mouse model with induced hearing loss, ensuring neuron function and survival.

One of the studies published in this SI investigated whether *Selaginella tamariscina* has an antiviral effect against influenza A virus (IAV) infection. They used green fluorescent protein (GFP)-tagged influenza A virus (IAV) to examine the effect of Selaginella tamariscina ethanol extract (STE) on influenza viral infection. Fluorescence microscopy and flow cytometry showed that STE potently represses the GFP expression of the virus in a dose-dependent manner. STE significantly inhibited the expression of the IAV M2, NP, HA, NA, NS1, and PB2 proteins. Time-of-addition and hemagglutination inhibition assays showed that STE showed an inhibitory effect on hemagglutinin and viral binding on the cells at an early infection stage. In addition, STE exerted a suppressive effect on the neuraminidase activity of the H1N1 and H3N2 IAVs. Furthermore, in a dose-dependent way, STE inhibited the cytopathic effect induced by H3N2, as well as by H1N1 IAV. Especially in the presence of STE, the cytopathic effect was completely blocked. Overall, these data suggest that STE has antiviral efficacy against IAV infection; thus, it could be developed as a natural IAV inhibitor.

The study that evaluated the antithrombotic action of *Acrocomia aculeata* pulp oil (AAPO) in natura in an in vitro experimental model reported that ADP/epinephrine induced platelet aggregation after treatment with AAPO, as evaluated using turbidimetry, and that coagulation was determined by the prothrombin activity time and activated partial thromboplastin time. Promising results showed that AAPO has major components such as oleic acid, palmitic acid, lauric acid, caprylic acid, and squalene. AAPO showed no toxicity in vitro or in vivo. Platelet aggregation decreased against agonists when using treatments with different concentrations of AAPO. The oil did not interfere with the prothrombin time or the activated partial thromboplastin time. Moreover, it expressly decreased ROS-induced platelet activation and P-selectin expression. Therefore, AAPO showed antiplatelet action, given that it decreased the platelet activation verified by the decrease in P-selectin expression as well as in ROS production.

The extract from the leaves of *Passiflora ligularis* (*P. ligularis*) showed significant effects on lowering serum glucose levels; it reduced insulin resistance, preserved pancreatic tissue, contributed antioxidant effects, and ameliorated the serum lipid profile in studies carried out in adult mice. Based on this experimental approach, the extract of *P. ligularis* exhibits a potential role in the treatment of type 2 diabetes. Finally, this SI discussed the multifaceted effects of extracts from plants on human health based on *Withania somnifera*, popularly known as Ashwagandha. The wide spectrum of action of this plant stems from its observed anti-inflammatory, neuroprotective, immunomodulatory, hepatoprotective, cardioprotective, anti-diabetic, adaptogenic, anti-arthritic, anti-stress, and antimicrobial effects.

The description above summarizes the updated data related to the extracts of plants and, in some cases, highlights specific extracts/plants characterized by major compounds in different disease models, such as in the prostate cancer cell line and C57BL/6 mouse prostate, rheumatoid arthritis, respiratory diseases, hyperuricemia, hearing loss, influenza A, thrombose, and diabetes, as well as reports on the multifaceted effects of extracts on inflammation, neuroprotection, immunomodulation, hepatoprotection, cardio protection, diabetes, arthritis, stress, and microbial factors. Therefore, we invite our readers who are passionate about this area to enjoy a pleasant read.

